# XBB.1.16‐RBD‐based trimeric protein vaccine can effectively inhibit XBB.1.16‐included XBB subvariant infection

**DOI:** 10.1002/mco2.687

**Published:** 2024-08-16

**Authors:** Dandan Peng, Cai He, Zimin Chen, Hong Lei, Xiya Huang, Chunjun Ye, Binhan Wang, Ying Hao, Xinyi Du, Shuaiyao Lu, Hongbo Hu, Wei Cheng, Haohao Dong, Jian Lei, Xikun Zhou, Xiangrong Song, Guangwen Lu, Xiawei Wei

**Affiliations:** ^1^ Laboratory of Aging Research and Cancer Drug Target, State Key Laboratory of Biotherapy and Cancer Center, National Clinical Research Center for Geriatrics West China Hospital Sichuan University Chengdu Sichuan China; ^2^ National Kunming High‐Level Biosafety Primate Research Center, Institute of Medical Biology Chinese Academy of Medical Sciences and Peking Union Medical College Kunming Yunnan China; ^3^ WestVac Biopharma Co. Ltd. Chengdu China

**Keywords:** heterologous booster, recombinant RBD‐HR protein, XBB.1.16 variant

## Abstract

The newly identified XBB.1.16‐containing sublineages, including XBB.1.5, have become the prevailing severe acute respiratory syndrome coronavirus 2 (SARS‐CoV‐2) variant in circulation. Unlike previous Omicron XBB variants (e.g., XBB.1.5 and XBB.1.9) harboring the F486P substitution, XBB.1.16 also carries a T478R substitution in the receptor‐binding domain (RBD). Numerous researchers have delved into the high transmissibility and immune evasion of XBB.1.16 subvariant. Therefore, developing a new vaccine targeting XBB.1.16, including variants of concern (VOCs), is paramount. In our study, we engineered a recombinant protein by directly linking the S‐RBD sequence of the XBB.1.16 strain of SARS‐CoV‐2 to the sequences of two heptad repeat sequences (HR1 and HR2) from the SARS‐CoV‐2 S2 subunit. Named the recombinant RBD_XBB.1.16_‐HR/trimeric protein, this fusion protein autonomously assembles into a trimer. Combined with an MF59‐like adjuvant, the RBD_XBB.1.16_‐HR vaccine induces a robust humoral immune response characterized by high titers of neutralizing antibodies against variant pseudovirus and authentic VOCs and cellular immune responses. Additionally, a fourth heterologous RBD_XBB.1.16_‐HR vaccine enhances both humoral and cellular immune response elicited by three‐dose mRNA vaccines. These findings demonstrate that the recombinant RBD_XBB.1.16_‐HR protein, featuring the new T478R mutation, effectively induces solid neutralizing antibodies to combat newly emerged XBB variants.

## Graphical abstract

The RBD_XBB.1.16_‐HR vaccine we formulated could elite high titers of receptor‐binding domain (RBD)‐specific binding antibodies, neutralizing antibodies against variants pseudovirus, and cellular immune responses. In addition, a heterologous RBD_XBB.1.16_‐HR vaccine could improve both RBD‐specific binding antibodies and neutralizing antibodies induced by Delta‐mRNA vaccines and BA.4/5‐mRNA vaccines. These data indicated that the recombinant RBDXBB.1.16‐HR protein with a new T478R mutation could induce strong neutralizing antibodies to fight newly emerged XBB variants

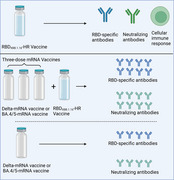

## 1 INTRODUCTION

Earlier in 2023, the XBB.1.16 variant emerged in India before spreading to other Western and Asian countries, marking a significant development in the trajectory of COVID‐19.^1^ Within a mere 4 weeks, its prevalence surged, leading to its classification as a variant of interest (VOI) on April 17, 2023.^2^ Stemming from the XBB.1.5 subvariant and characterized by the F486P substitution in the receptor‐binding domain (RBD), XBB.1.16 also harbors a T478R substitution in the RBD. Extensive research has investigated its high transmissibility and immune evasiveness, with its adequate reproductive number being 1.22‐ and 1.13‐fold higher than XBB.1 and XBB.1.5, respectively.^3^, ^4^ Despite a lower binding affinity of XBB.1.16 RBD to the human angiotensin‐converting enzyme 2 (ACE2) receptor compared to XBB.1.5, the profound immune evasion of XBB.1.16 was similar to XBB.1.5.^4^ These findings highlight the urgent need to develop a new vaccine targeting newly emerged variants like XBB.1.16. Additionally, the World Health Organization (WHO) supports creating monovalent vaccines targeting only one strain, underscoring the urgency of developing a monovalent vaccine to combat newly emerged variants and prevent immune escape.^5^

People in many countries have already been immunized with the mRNA COVID‐19 vaccine (mRNA‐1273 and mRNA BNT162b2).^6^, ^7^ However, despite their efficacy at preventing infection by the ancestral strain, their antibody response to BA.4/5‐included Omicron sublineages has been limited.^8^ To address the challenge, bivalent COVID‐19 vaccines were designed, combining the spike of Wuhan‐1 with either BA.1 (mRNA‐1273.214) or BA.4/5 (mRNA‐1273.222). These bivalent COVID‐19 vaccines have demonstrated the ability to broadly neutralize antibodies and enhance protection against BA.4/5 viruses in mice and clinical trials.^9^ Moreover, booster vaccines of both mRNA‐1273.214 and mRNA‐1273.222 have shown promise in providing additional protection against BA.4/5‐included Omicron sublineages,^10^, ^11^, ^12^ with mRNA‐1273.222 being particularly effective in preventing COVID‐19‐related outpatient visits and hospitalizations in older adults (≥65).^13^ Subsequently, the food and drug administration (FDA) authorized using the mRNA‐1273.222 vaccine as a booster dose. Furthermore, research has investigated the efficacy of using the RBD_XBB.1.16_‐HR vaccine as a heterologous booster.

**FIGURE 1:**
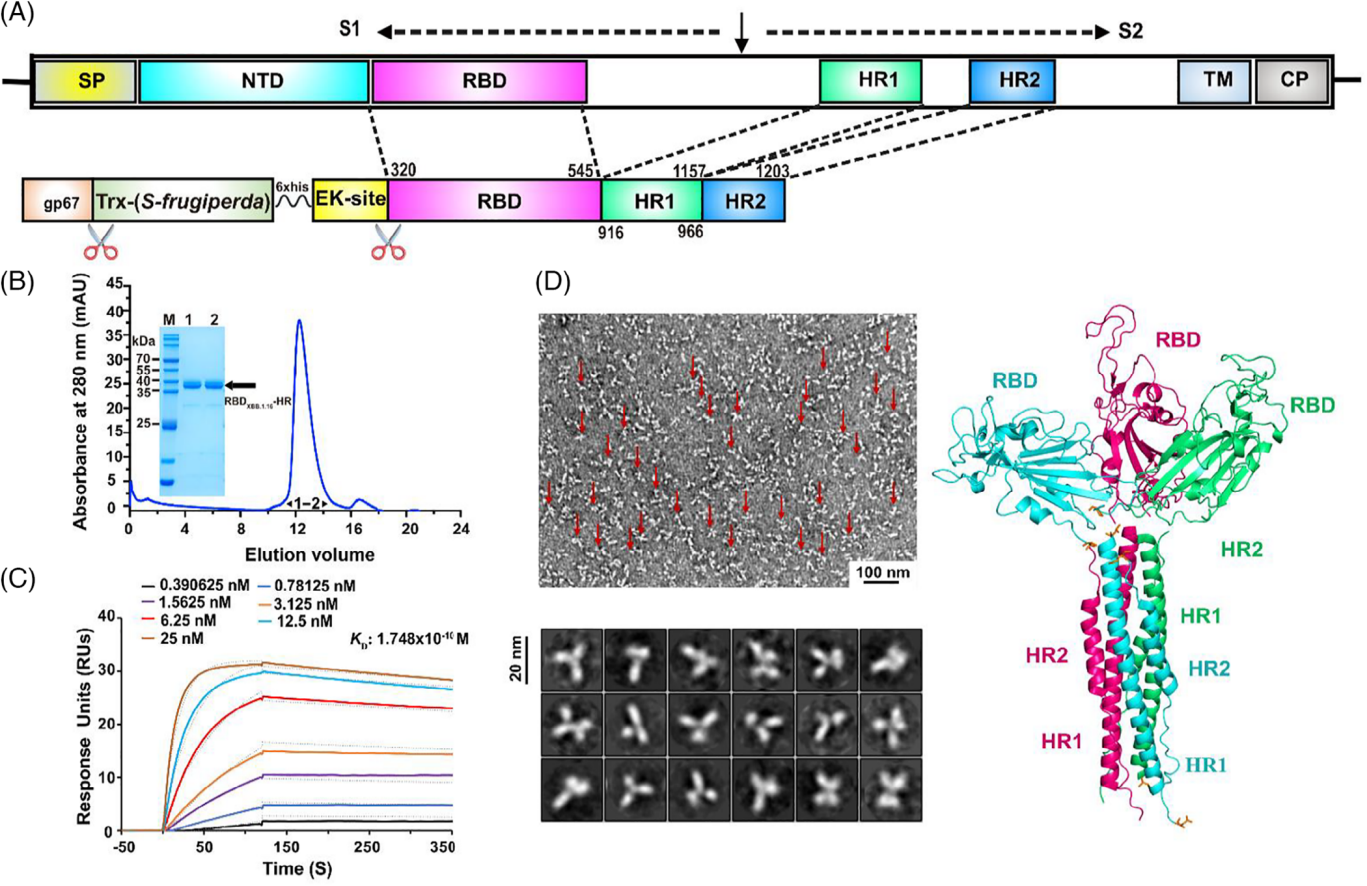
Characteristic of the trimeric receptor‐binding domain (RBD)_XBB.1.16_‐HR recombinant protein. (A) The construct design of the XBB.1.16‐RBD‐based trimeric protein. Our trimeric RBD_XBB.1.16_‐HR recombinant protein features with the direct linkage of the S‐RBD sequence of the severe acute respiratory syndrome coronavirus 2 (SARS‐CoV‐2) XBB.1.16 strain (amino acids 320–545) to an HR1 (amino acids 916–966) and an HR2 sequence (amino acids 1157–1203) of the SARS‐CoV‐2 S2 subunit. CP, cytoplasmic domain; HR1 and HR2, heptad repeats 1 and 2; NTD, N‐terminal domain; RBD, receptor‐binding domain; SP, signal peptide; TM, transmembrane domain. (B) A representative elution chromatograph of the RBD_XBB.1.16_‐HR recombinant protein using a Superdex 200 Increase 10/300 GL column (GE Healthcare). The sodium dodecyl sulfate‐polyacrylamide gel electrophoresis (SDS‐PAGE) of the RBD_XBB.1.16_‐HR recombinant protein was shown. (C) Surface plasmon resonance (Biacore) performed the real‐time protein binding kinetics. (D) Transmission electron micrographs (left) and molecular model (right) of the trimeric RBD_XBB.1.16_‐HR recombinant protein with a bundle‐like shape.

Our study developed a new severe acute respiratory syndrome coronavirus 2 (SARS‐CoV‐2) vaccine based on the RBD of the XBB.1.16 variant and specific components of the SARS‐CoV‐2 S2 subunit (HR1 and HR2; Figure 1). This vaccine, termed RBD_XBB.1.16_‐HR, self‐assembles as a trimeric recombinant protein.^14^ Following intramuscular immunization, the RBD_XBB.1.16_‐HR vaccine (recombinant RBD_XBB.1.16_‐HR protein + MF59‐like adjuvant) has been found to elicit and evaluate robust humoral and cellular immunity. Notably, broadly pseudovirus‐neutralizing and virus‐neutralizing antibodies indicate its potential to fight infections caused by variants of concern (VOCs).

Furthermore, the potential of the RBD_XBB.1.16_‐HR vaccine as a heterologous sequential booster following mRNA vaccine priming has been explored. After three doses of mRNA vaccine, a fourth dose of the heterologous RBD_XBB.1.16_‐HR boosting vaccine has shown potential to enhance vaccine‐induced humoral and cellular immunity. In conclusion, studies suggest that the RBD_XBB.1.16_‐HR vaccine, incorporating the new T478R mutation, could induce neutralizing antibodies capable of combating newly emerged XBB subvariants.

## 2 RESULTS

### 2.1 Construction and characterization of the recombinant RBD_XBB.1.16_‐HR protein

The construct design is illustrated in Figure 1A. We essentially engineered an RBD_XBB.1.16_‐HR protein compromising an RBD (320–545 aa) sequence derived from the XBB.1.16 Omicron subvariant, along with HR1 and an HR2 sequences derived from the SARS‐CoV‐2 S2 subunit, fused in tandem. Subsequently, the protein antigen was expressed using insect cells and the Bac‐to‐Bac baculovirus expression system, as previously outlined.^14^, ^15^ Following purification, we successfully obtained the RBD_XBB.1.16_‐HR protein with over 98% purity and good homogeneity. Its formation was verified through gel‐filtration chromatography and sodium dodecyl sulfate‐polyacrylamide gel electrophoresis (SDS‐PAGE), as depicted in Figure 1B. Additionally, we measured the binding interaction between our protein and human ACE2, which serves as the cellular receptor for SARS‐CoV‐2, through surface plasmon resonance (SPR) analysis. As anticipated, the kinetic binding data revealed an equilibrium dissociation constant (*K*_D_) of 0.1748 nM (Figure 1C), coinciding with our prior findings and those of others.^16^ Additionally, negative‐staining transmission electron microscopy (TEM) images of negative‐stained samples confirmed the assembly of the protein into a trimeric structure, akin to the protein assembly mode depicted in the panel on the right (Figure 1D).

### 2.2 RBD_XBB.1.16_‐HR vaccine induces broad‐spectrum neutralizing antibodies against EG.5.1‐included VOCs

**FIGURE 2:**
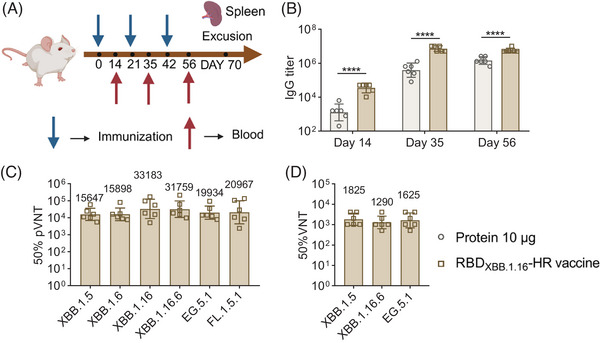
Receptor‐binding domain (RBD)_XBB.1.16_‐HR‐induced humoral immune responses in serum. (A) Scheme of experiments. Six to eight‐week‐old NIH female mice were immunized with the RBD_XBB.1.16_‐HR vaccine via an intramuscular route on days 0, 21, and 42. Sera samples were collected on days 14, 35, and 56. Serum on day 56 was used to measure the pseudovirus neutralization test and virus neutralization test (VNT). Immunized mice were excused on day 70, and the humoral and cellular immunization responses of the spleen were detected. (B–D) Humoral immunity response in serum of immunized mice. RBD‐specific immunoglobulin G (IgG) antibodies (B) and neutralizing antibodies against pseudovirus (C) and authentic variants of concern (VOCs) (D). (A) was created by BioRender, (B–D) were prepared by GraphPad 9.0. Bars and columns show geometric mean with geometric standard deviation (SD) values. *p* values in (B) were determined by two‐way analysis of variance (ANOVA) with compare each cell mean with the other cell mean in that row. *p < 0.05; **p < 0.01; ***p < 0.001; ****p < 0.0001; ns, not significant.

Six to eight‐week‐old female national institutes of health (NIH) mice (*n* = 6) was immunized with a range of doses of RBD_XBB.1.16_‐HR vaccine (1 µg: RBD_XBB.1.16_‐HR_low_; 5 µg: RBD_XBB.1.16_‐HR_middle_; 10 µg: RBD_XBB.1.16_‐HR_high_). As previously observed and reported,^14^, ^15^ a dose of 10 µg induced the highest titers of neutralizing antibodies (Supporting Information Figure 1). Therefore, we chose an appropriate dose (10 µg) in subsequent research. As controls, mice were administrated phosphate buffered saline (PBS) or protein without adjuvant intramuscularly on days 0, 21, and 42 (Figure 2A). Blood samples were collected on days 14, 35, and 56, and spleens were harvested for detection of cellular immune response detection. Binding antibodies against RBD (XBB.1.16) were measured using an enzyme‐linked immunosorbent assay (ELISA) for all selected serum.

Three doses of RBD_XBB.1.16_‐HR vaccine induced high levels of RBD‐specific immunoglobulin G (IgG) antibody titers (Figure 2B). Notably, the RBD_XBB.1.16_‐HR protein, even without MF59‐like adjuvant, induced significantly lower RBD‐specific IgG antibodies at levels comparable to those observed with the MF59‐like‐adjuvanted vaccine. The geometric mean titers (GMTs) of the 50% pseudovirus neutralization test (50% pVNT) against XBB.1.5, XBB.1.6, XBB.1.16, XBB.1.16.6, EG.5.1, and FL.1.5.1 were 15,647, 15,898, 33,183, 31,759, 19,934, 20,967, respectively (Figure 2C). Neutralizing antibodies in sera against authentic viruses were detected. As expected, three doses of RBD_XBB.1.16_‐HR induced high levels of 50% virus neutralization test (50% VNT) against authentic XBB.1.5, XBB.1.16.6, and EG.5.1, with GMTs at 1825, 1290, and 1625, respectively (Figure 2D). These findings highlight the ability of the RBD_XBB.1.16_‐HR vaccine to induce and elicit high levels of humoral immune responses for combating SARS‐CoV‐2 infection.

### 2.3 RBD_XBB.1.16_‐HR vaccine elicited robust cellular immune responses in mice spleen

**FIGURE 3:**
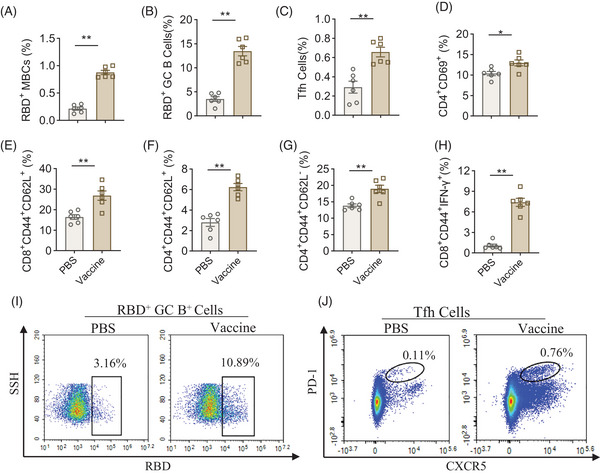
Receptor‐binding domain (RBD)_XBB.1.16_‐HR vaccine‐induced T cell responses in spleen. (A–C) Spleen RBD‐specific memory B cells (MBCs), germinal center (GC) B cells, and follicular helper T (Tfh) cells frequencies on day 70. (D) Spleen activated CD4^+^ T cell frequencies on day 70. (E, F) Spleen CD4^+^ central memory T cell frequencies on day 70. (G) Effector memory frequencies in the vaccinated spleen on day 70. (H) XBB.1.5 spike‐specific CD8^+^CD44^+^IFN‐γ^+^ T cell response on day 70. (I, J) The original flow cytometry data of RBD‐specific GC B cells and effector Tfh cells. Data represent one independent experiment. Data shown are geometric mean with geometric standard deviation (SD) values. *p* values were conducted by unpaired two‐tailed Mann–Whitney test. *p < 0.05; **p < 0.01; ***p < 0.001; ****p < 0.0001; ns, not significant.

We analyzed T cell and B cell responses in the spleens of vaccinated animals on day 70 (28 days after the third dose) using flow cytometry. Consistent with the previous studies,^14^, ^17^ the RBD_XBB.1.16_‐HR vaccine exhibited robust germinal center (GC) responses, characterized by elevated levels of RBD (XBB.1.16)‐specific memory B cells (MBCs) and RBD‐specific GC B cells and follicular helper T (Tfh) cells (Figure 3A–C). Regarding T cell responses, we assessed the populations of activated T cells, central memory T cells, and effector memory T cells via flow cytometry. Furthermore, we analyzed RBD‐specific IFN‐γ‐secreting T cells using an intracellular cytokine staining (ICS) assay. Our finding revealed increased CD4^+^ activated T cells in the RBD_XBB.1.16_‐HR vaccine group (Figure 3D). Moreover, the frequencies of CD8^+^ and CD4^+^ central memory T cells were significantly elevated compared to the control group (Figure 3E,F), along with increased levels of CD4^+^ effector memory T cells (Figure 3G). The ICS assay demonstrated a significant increase in IFN‐γ‐secreting CD8^+^ cells in the RBD_XBB.1.16_‐HR vaccine group (Figure 3H). These results collectively indicated that the RBD_XBB.1.16_‐HR vaccine could elicit a well‐regulated GC center immune response and T cell immune response, which may persist over time and contribute to establishing memory immune responses.

### 2.4 RBD_XBB.1.16_‐HR heterologous booster improves humoral immune responses against XBB lineages

**FIGURE 4:**
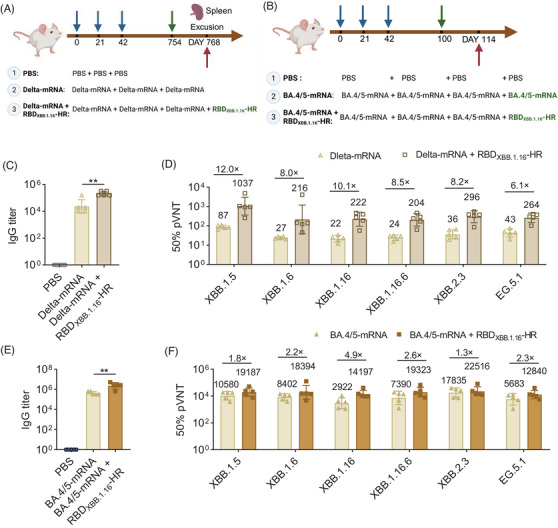
Receptor‐binding domain (RBD)_XBB.1.16_‐HR heterologous booster vaccine after three‐dose priming mRNA vaccine elicits stronger humoral immunity. Scheme of heterologous booster on BA.4/5‐mRNA (A) and Delta‐mRNA (B) vaccine experiments. The immunoglobulin G (IgG) titer (C) and neutralization antibodies (D) on BA.4/5‐mRNA heterologous vaccine. The IgG titer (E) and neutralization antibodies (F) on Delta‐mRNA heterologous vaccine. (A) and (B) were created by BioRender. Data shown are geometric mean with geometric standard deviation (SD) values. *p* values were determined by one‐way analysis of variance (ANOVA) followed by Tukey's multiple comparisons test. *p < 0.05; **p < 0.01; ***p < 0.001; ****p < 0.0001; ns, not significant.

To assess the potential of the RBD_XBB.1.16_‐HR booster vaccine in aged models (mouse >18 months),^18^ we conducted a study using NIH female mice that had received a three‐dose Delta‐mRNA vaccine, followed by administration of the fourth booster or no booster (Figure 4A). In line with clinical trial data demonstrating high titers of S‐specific antibodies and cellular immunity induced by two doses of mRNA‐1273, BNT162b2, and ChAdOx1 after 6–9 months,^19^ we administrated the fourth booster 24 months after the third Delta‐mRNA vaccine. Serum samples and tissues were collected 14 days after the fourth vaccine. Notably, comparable RBD‐specific antibody titers were observed, with a GMT at 22,974, while the 3 × Delta‐mRNA + 1 × RBD_XBB.1.16_‐HR heterologous group, exhibited higher RBD‐specific antibody titers, with GMT at 211,121 (Figure 4C). Consistent with previous findings,^20^ three doses of Delta‐mRNA vaccine resulted in comparable neutralization titers against XBB.1.5, XBB.1.6, XBB.1.16, XBB.1.16.6, XBB.2.3, EG.5.1 pseudovirus strains with GMTs at 87, 27, 22, 24, 36, 43 two years postimmunization, respectively. Notably, the GMTs in the three doses Delta‐mRNA + a dose of RBD_XBB.1.16_‐HR group showed improvements by factors of 12.0×, 8.0×, 10.1×, 8.5×, 8.2×, and 6.1×, respectively (Figure 4D). These results suggested that the RBD_XBB.1.16_‐HR vaccine holds promise as a potential booster to enhance broad‐spectrum neutralizing antibodies against XBB sublineages.

In the case of the BA.4/5‐mRNA vaccine, Female NIH mice (*n* = 5) were immunized with three doses of the BA.4/5‐mRNA vaccine on days 0, 21, and 42. Subsequently, on day 100, they were administered either a fourth homologous BA.4/5‐mRNA vaccine or a heterologous RBD_XBB.1.16_‐HR vaccine on day 100. Sera were gathered on day 114, and humoral immune responses were assessed (Figure 4B). The heterologous group elicited high levels of IgG antibodies, with the GMT of IgG antibody titers being 5.3‐fold more than the homologous group (Figure 4E). Additionally, the GMTs of 50% pVNT against XBB.1.5, XBB.1.6, XBB.1.16, XBB.1.16.6, XBB.2.3 and EG.5.1 in the homologous BA.4/5‐mRNA group were 10,580, 8402, 2922, 7390, 17,835, and 5683, respectively. Notably, the GMTs of 50% pVNT against the pseudovirus mentioned above strains in the heterologous group were 1.8‐, 2.2‐, 4.9‐, 2.6‐, 1.3‐, and 2.3‐fold than the homologous group (Figure 4F).

### 2.5 XBB.1.16 heterologous booster vaccine enhances the cellular immune response after priming three‐dose Delta‐mRNA vaccine

**FIGURE 5:**
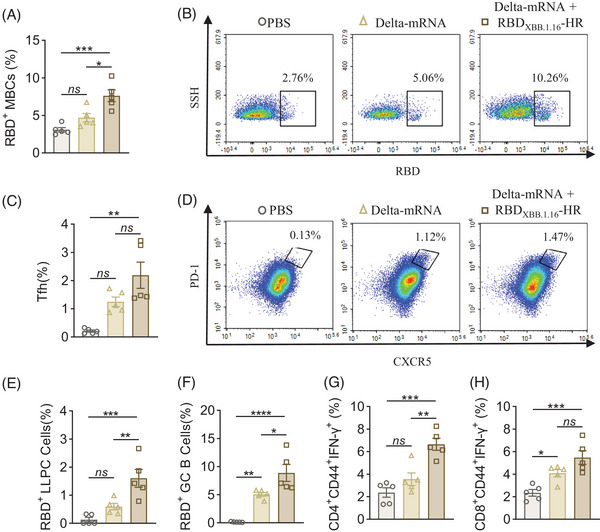
Cellular immune response after the fourth heterologous receptor‐binding domain (RBD)_XBB.1.16_‐HR booster. (A, C, E, and F) Spleen RBD‐specific memory B cells (MBCs), effector follicular helper T (Tfh) cells, long‐lived plasma cells (LLPCs), germinal center (GC) B cells frequencies. (B, D) The original flow cytometry data of RBD‐specific MBCs and effector Tfh cells. (G, H) XBB.1.5 spike‐specific CD4^+^CD44^+^IFN‐γ^+^ and CD8^+^CD44^+^IFN‐γ^+^ T cell response in spleen. Bars and columns show the mean with SEM. *p* values were determined by one‐way analysis of variance (ANOVA) followed by Tukey's multiple comparisons test. *p < 0.05; **p < 0.01; ***p < 0.001; ****p < 0.0001; ns, not significant.

For cellular immune responses, three doses of Delta‐mRNA vaccine induced strong frequencies of GC B cells and CD8^+^CD44^+^IFN‐γ^+^ in the vaccinated spleen for at least 2 years, while other cellular responses did not significantly differ from those in the PBS group. These findings underscore the need for a SARS‐CoV‐2 vaccine booster. In our study, the fourth dose of RBD_XBB.1.16_‐HR vaccine effectively recalled and enhanced the priming memory immune responses, including MBCs (Figure 5A,B) and long‐lived plasma cells (LLPCs) (Figure 5E). Although the frequency of Tfh cells did not significantly improve (Figure 5C,D), there was a notable elevation in RBD‐specific GC B cells (Figure 5F). These data indicated that three‐dose Delta‐mRNA could induce a high level of central memory response for at least 2 years, and fourth‐dose protein vaccine could activate the memory immune response. In terms of ICS, the frequency of CD4^+^CD44^+^IFN‐γ^+^ in the heterologous group was higher than in the Delta‐mRNA group (Figure 5G), while the frequency of CD8^+^CD44^+^IFN‐γ^+^ cells was similar (Figure 5H). These results indicated that three doses of the Delta‐mRNA vaccine could induce a comparable immune response against SARS‐CoV‐2 for at least 2 years. Moreover, a fourth RBD_XBB.1.16_‐HR booster could reactivate Delta‐mRNA‐induced memory immune responses and enhance both humoral and cellular immune responses.

## 3 DISCUSSION

Considering the ongoing emergence of SARS‐CoV‐2 variants aimed at evading immune responses, updating COVID‐19 vaccine compositions based on newly emerged viruses is necessary.^21^ Developing a recombinant protein vaccine based on XBB.1.16 is crucial. In this study, we have developed a recombinant protein vaccine utilizing the RBD of the XBB.1.16 variant and formulated it with an MF59‐like adjuvant (RBD_XBB.1.16_‐HR vaccine), as previously described.^14^ As expected, the RBD_XBB.1.16_‐HR vaccine induced robust humoral and cellular immune responses. Administration of the RBD_XBB.1.16_‐HR vaccine resulted in elevated levels of neutralizing antibodies against pseudoviruses (including XBB.1.5, XBB.1.6, XBB.1.16, XBB.1.16.6, and EG.5.1) and authentic viruses (XBB.1.5, XBB.1.16.6, EG.5.1), along with GC immune response and increased IFN‐γ‐secreting CD8^+^ T cells.

In a previous study, we demonstrated that following two‐dose priming with the Delta‐mRNA vaccine, a third heterologous RBD‐HR (Delta) vaccine booster 3 months apart could induce more robust immune responses than a third homologous Delta‐mRNA vaccine.^22^ In this study, we administrate the fourth booster (RBD_XBB.1.16_‐HR vaccine or PBS) 2 years after the third‐dose Delta‐mRNA vaccine. Our data indicated that three doses of Delta‐mRNA vaccine could still induce a comparable humoral immune response and cellular immune response. The fourth RBD_XBB.1.16_‐HR booster significantly stimulated the humoral and cellular immune responses.

Compared with Delta mutant strains, BA.4/5‐included Omicron sublineages have more substantial immune evasion capabilities.^23^ Fortunately, a bivalent BA.4/5‐based mRNA vaccine (mRNA1273.222) has been authorized for booster vaccine use. Similar to mRNA1273.222,^9^ our data also showed that four doses of BA.4/5‐mRNA vaccine induced potent neutralizing antibodies against XBB.1.5, XBB.1.6, XBB.1.16, XBB.1.16.6, XBB.2.3, and EG.5.1 pseudoviruses. However, a fourth booster vaccine, RBD_XBB.1.16_‐HR, elicited more excellent broad‐spectrum neutralizing antibodies than the homologous BA.4/5 mRNA vaccine. However, we acknowledge that our study did not analyze the difference in immunogenicity between the BA.4/5‐mRNA vaccine induced by our method and other mRNA vaccines commercially available. These aspects represent important considerations for future research to comprehensively evaluate the effectiveness and comparative immunogenicity of different vaccine formulations and priming strategies in combating emerging SARS‐CoV‐2 variants. Such investigations would contribute valuable insights to optimize vaccination strategies and enhance global efforts to control the COVID‐19 pandemic.

In conclusion, developing vaccines targeting newly emerged SARS‐CoV‐2 variants, such as the XBB.1.16 subvariant, is crucial in the ongoing battle against the COVID‐19 pandemic. Our study demonstrates the potential of the recombinant RBD_XBB.1.16_‐HR vaccine to induce robust humoral and cellular immune responses against XBB sublineages, including those with increased immune evasion capabilities. Furthermore, our findings suggest that incorporating this vaccine as a booster shot could enhance immunity in individuals who have received previous doses of mRNA vaccines. While further research is needed to fully understand the efficacy and safety of the RBD_XBB.1.16_‐HR vaccine in human populations, our study provides promising evidence for its potential role in combating the evolving landscape of SARS‐CoV‐2 variants.

## 4 METHODS

### 4.1 Cell lines

293T/ACE2 cells^17^ were cultured in Dulbecco's modified Eagle's medium (DMEM, Gibco) supplemented with 10% fetal bovine serum (FBS, Gibco) and 100 mg/mL penicillin–streptomycin at 37°C with 5% CO_2_. Sf9 cells (ATCC CRL‐1711)^14^ were sustained in SIM SF medium (Sino Biological) on a nonhumidified shaker at 28°C.

### 4.2 Protein expression and characterization

The RBD_XBB.1.16_‐HR protein was expressed in *Spodoptera frugiperda* Sf9 cells by Bac‐to‐Bac baculovirus expression system (Invitrogen) as previously described.^24^ Briefly, our construct design involved directly linking the S‐RBD sequence of the SARS‐CoV‐2 XBB.1.16 strain (amino acids 320–545) to sequences for HR1 and HR2 of the SARS‐CoV‐2 S2 subunit. For protein production, the coding sequences including a GP67 signal peptide, a Trx tag, a 6xHis tag, and an Enterokinase (EK) cleavage site were further fused into the N‐terminus for protein secretion, folding, purification, and tag removal, respectively.^25^

These gene fragments were initially amplified and then subcloned into pFastbac1 vector. Each sequencing‐verified recombinant plasmid was transformed into *Escherichia coli* DH10b cells to produce recombinant bacmids, which were subsequently transfected into sf9 cells for protein production. Then, the protein binding affinities were detected by SPR assay, and the characterization of protein formations was identified through TEM imaging.^26^, ^27^, ^28^, ^29^

### 4.3 Vaccine formulation

We formulated the RBD_XBB.1.16_‐HR as previously.^14^ Briefly, the purified RBD_XBB.1.16_‐HR protein was diluted in the MF59‐like adjuvants in equal volumes to form vaccines with 200 µg/mL protein.

Delta‐mRNA vaccines were formulated as before.^22^ The formulation of the BA.4/5‐mRNA vaccine was similar to the Delta‐mRNA vaccine unless the BA.4/5‐mRNA vaccine expressed the full length of the spike protein of the BA.4/5 variant.

### 4.4 Mouse vaccination

The female NIH mice (6–8 weeks) were purchased from Beijing Vital River Laboratory Animal Technology Co., Ltd. and maintained in the animal center of the State Key Laboratory of Biotherapy, Sichuan University.

To explore the immunogenicity of RBD_XBB.1.16_‐HR vaccines, NIH mice were intramuscularly immunized with PBS, purified RBD_XBB.1.16_‐HR recombinant protein, or RBD_XBB.1.16_‐HR vaccination on days 0, 21, and 42. The vaccinated serum samples were collected on day 14, 35, and 56. To assay the cellular immune response induced by the RBD_XBB.1.16_‐HR vaccine, the spleen of the vaccinated mice were collected and analyzed by flow cytometry.

To evaluate the potential of the RBD_XBB.1.16_‐HR vaccine as a heterologous booster, the NIH mice (6–8 weeks) were intramuscularly immunized with two kinds of mRNA vaccines on day 0, 21, and 42. mRNA vaccine expresses the full‐length S protein of the BA.4/5 variant. After three doses of BA.4/5‐mRNA vaccine, a fourth homologous BA.5‐mRNA vaccine (5 µg) or a heterologous RBD_XBB.1.16_‐HR vaccine (10 µg) were immunized on day 100, serum and spleen were collected 14 days later after the fourth vaccination.

To investigate the effect of RBD_XBB.1.16_‐HR vaccine booster on memory immune responses induced by previous vaccines, the NIH mice which were immunized with three doses of Delta‐mRNA^22^ (5 µg) on days 0, 21, and 42, were vaccinated with a fourth dose of RBD_XBB.1.16_‐HR vaccine (10 µg) on day 600 (3 × Delta‐mRNA + 1 × RBD_XBB.1.16_‐HR). As a control, mice were immunized with three doses of Delta‐mRNA on days 0, 21, and 42 (3 × Delta‐mRNA). The serum, spleen, and bone marrow lymphocytes of both Delta‐mRNA groups were collected on day 614.

### 4.5 Enzyme‐linked immunosorbent assays

Purified recombinant trimeric protein RBD_XBB.1.16_‐HR was diluted to 1 µg/mL in carbonate coating buffer (50 mM, pH 9.6) and coated onto a 96‐well plate (NUNC‐MaxiSorp, Thermo Fisher Scientific) overnight (100 µL/well). The plate was then washed three times with PBS containing 0.1% Tween‐20 (PBST) and blocked with 200 µL PBST containing 1% bovine serum albumin (BSA) for 1 h at room temperature. After washed, the plates were added with serially diluted sera (100 µL) and coated at room temperature. An hour later, the plates were washed with PBST more than three times and coated with diluted horseradish peroxidase (HRP)‐conjugated anti‐mouse IgG antibodies (Invitrogen, 31430) for an hour at 37°C. Following five washes with PBST, the plates were incubated with 100 µL of 3,3′,5,5′‐tetramethylbenzidine (TMB, Thermo Fisher Scientific, 34029) per well for 10 min in the dark. The reaction was stopped by adding 100 µL/well Stop Solution for TMB Substrate (Beyotime, P0215), and the absorbance at 450 nm was measured using a microplate reader (Spectramax ABS, Molecular Devices) equipped with SoftMax Pro 7.1 software.

### 4.6 Pseudovirus neutralization assay

Several newly emerged pseudoviruses (Genomeditech) were used to explore RBD_XBB.1.16_‐HR vaccine‐induced broadly neutralizing antibodies. Briefly, threefold diluted sera (50 µL) were coincubated for an hour with 50 µL of diluted pseudovirus. Then, 1.2 × 10^4^ 293T/ACE2 cells (100 µL) were added to each well and incubated for 48 h. Wells without serum were seen as a negative control, and wells without pseudoviruses were seen as a positive control. Remove the supernatant and add 100 µL lysis reagent with luciferase substrate (Beyotime, RG005) to each well. The relative light unit (RLU) was assessed using a multimode microplate reader (PerkinElmer) with Kaleido 3.0 software, and analysis was conducted using GraphPad Prism 9.0.^14^

### 4.7 Live SARS‐CoV‐2 virus neutralization assay

An authentic virus neutralization assay was established to detect vaccinated sera's neutralizing activity and inhibitory ability against emerged VOCs. In short, diluted serum samples were coincubated with live SARS‐CoV‐2 virus at 50% tissue‐culture infectious dose (TCID50) for an hour at 37°C before adding them to Vero cells (ATCC CCL‐81) preplated at a density of 5 × 10^4^ cells/well in 96‐well culture plates. After 72 h, using a microscope, the neutralizing titers of vaccinated sera, resulting in 50% neutralization, were determined by observing cytopathogenic effects (CPEs).

### 4.8 Flow cytometry

Spleen and bone marrow were harvested to detect vaccine‐induced cellular immune responses and long‐term memory immune responses. Cells were incubated in 100 µL cold PBS containing 1% BSA for antibody staining. Cells in spleens were stained with fluorochrome‐conjugated antibodies against mouse: CD3‐PerCP/Cyanine5.5 (BioLegend, 100718), CD4‐APC (BioLegend, 100412), CD8‐FITC (BioLegend, 100706), CD44‐BV510 (BioLegend, 103044), CD69‐PE (BioLegend, 164204), CD62L‐BV421 (BioLegend, 104436), CD19‐BV421 (BioLegend, 115538), CXCR5‐PE (BioLegend, 145504), and PD‐1‐BV510 (BioLegend, 135241). For RBD‐specific cell staining, cells in spleens/bone marrow were incubated with biotinylated SARS‐CoV‐2 RBD protein (Acro, SPD‐C82Q4) at room temperature for 40 min. Washed twice, then stained on ice with the PE Streptavidin (BioLegend, 405204), B220‐PerCP/Cyanine5.5 (BioLegend, 103236), IgD‐BV510 (BioLegend, 405723), CD19‐FITC (BD Pharmingen™, 553785), CD38‐PE/Cy7 (BioLegend, 102718), GL‐7‐APC (BioLegend, 144618), CD4‐PerCP/Cyanine5.5 (BioLegend, 100540), CD44‐BV510 (BioLegend, 103044), CXCR5‐APC (BioLegend, 145506), CD45‐PerCP/Cyanine5.5 (BioLegend, 103132), CD138‐BV421(BioLegend, 142508), B220‐FITC (BioLegend, 103205) for 30 min. Gating strategy is shown in Supporting Information Figures 2–4. Activated T cells (CD4^+^CD69^+^, CD8^+^CD69^+^),^30^ CD4^+^ and CD8^+^ central memory (CD44^+^CD62L^+^) T cells and effector memory (CD44^+^CD62L^‐^) T cells,^31^ MBCs (B220^+^CD19^+^IgD^−^GL7^−^CD38^+^), GC B (CD4^−^CD19^+^CD95^+^GL7^+^) cells, Tfh (CD3^+^CD19^−^CD4^+^CXCR5^+^PD‐1^+^) cells, and LLPC (CD19^−^CD45^+^B220^−^CD138^+^) cells were measured using NovoExpress 1.4.1 software after measurements were taken with the NovoCyte Flow Cytometer (ACEA Biosciences).

The ICS assays were used to measure the functional response of S‐specific CD4^+^ and CD8^+^ cells. In brief, 1.5 × 10^6^ lymphocytes in spleens incubated in 1 mL 1640 medium with 10% FBS, 100 µg/mL streptomycin, 100 U/mL penicillin, 1 mM pyruvate (all from Gibco), 50 µM β‐mercaptoethanol, and 20 U/mL IL‐2 (all from Sigma‐Aldrich) were stimulated with 1 µg/mL peptide pools of XBB.1.16 (Vazyme Biotech Co., Ltd). Six hours later, 1 µL Golgi Plug protein transport inhibitor containing brefeldin A were added to each well and samples were incubated for 4 more h. The surface of cells stained with Live/Dead‐APC‐Cy7 (Invitrogen™, L34976), CD3‐PerCP/Cyanine5.5, CD4‐APC, CD4‐BV421 (BioLegend, 100438), CD8‐FITC, CD44‐BV510. Washed twice, cells were suspended with 200 µL fixation/permeabilization (BD Bioscience, 554715) solution on ice for 30 min. Then cells were washed and suspended with 1 × Perm/Wash™ Buffer and stained with IFN‐γ‐PE (BioLegend, 554412) or IFN‐γ‐BV421 (BioLegend, 505830). Flow cytometry data were obtained using the NovoCyte Flow Cytometer (ACEA Biosciences) and subsequently analyzed utilizing NovoExpress 1.4.1 software.

### 4.9 Statistical analysis

Statistical analyses were conducted using Prism software (GraphPad Prism 8.0). Two‐way analysis of variance (ANOVA) was employed to compare each cell mean with the other cell means in that row for two multivariate groups. The Mann–Whitney test was employed to compare two univariate groups. Comparisons involving three or more groups utilized one‐way ANOVA followed by Tukey's multiple comparisons test. *p < 0.05; **p < 0.01; ***p < 0.001; ****p < 0.0001; ns, not significant.

## AUTHOR CONTRIBUTIONS

Xiawei Wei, Guangwen Lu conceived and supervised, and designed the experiments. Xiangrong Song performed the Delta‐mRNA and BA.4/5‐mRNA vaccine. Dandan Peng, Cai He, and Zimin Chen performed protein vaccine formulation and vaccinations in animals. Dandan Peng, Cai He, Xiya Huang, and Xinyi Du performed a binding antibodies assay and pseudovirus neutralization experiment. Dandan Peng, Cai He, Hong Lei, Xiya Huang, Chunjun Ye, Binhan Wang, and Ying Hao collected tissues and performed flow cytometry to assay cellular immune responses. Dandan Peng and Cai He analyzed the data and wrote the manuscript. All the authors have read and approved the final manuscript.

## CONFLICT OF INTEREST STATEMENT

This work was supported by the WestVac Biopharma Co. Ltd. Guangwen Lu and Xiawei Wei, who are also working at the WestVac Biopharma Co. Ltd. The remaining authors declare no conflicts of interest.

## ETHICS STATEMENT

All animal studies followed and approved by the Institutional Animal Care and Use Committee of Sichuan University (Chengdu, Sichuan, China) with approval numbers at 20210409042 and 20230307025.

## Supporting information

Supporting Information

## Data Availability

All the data from the corresponding authors are available upon reasonable request.
