# Supplementary material for: XBB.1.16‐RBD‐based trimeric protein vaccine can effectively inhibit XBB.1.16‐included XBB subvariant infection
Source: MedComm (2020). 2024 Aug 16;5(9):e687. doi: 10.1002/mco2.687 (PMC11329747; doi:10.1002/mco2.687)
Supplement: Supplementary file 1 — Supporting Information [file MCO2-5-e687-s001.docx]

**Supplemental information**

**XBB.1.16-RBD-based trimeric protein vaccine can effectively inhibit XBB.1.16-included XBB subvariant infection**

Correspondence to: Xiawei Wei, [xiaweiwei@scu.edu.cn](mailto:xiaweiwei@scu.edu.cn) (Editorial corresponding author)

**Includes:**

Supplemental Figure 1-4


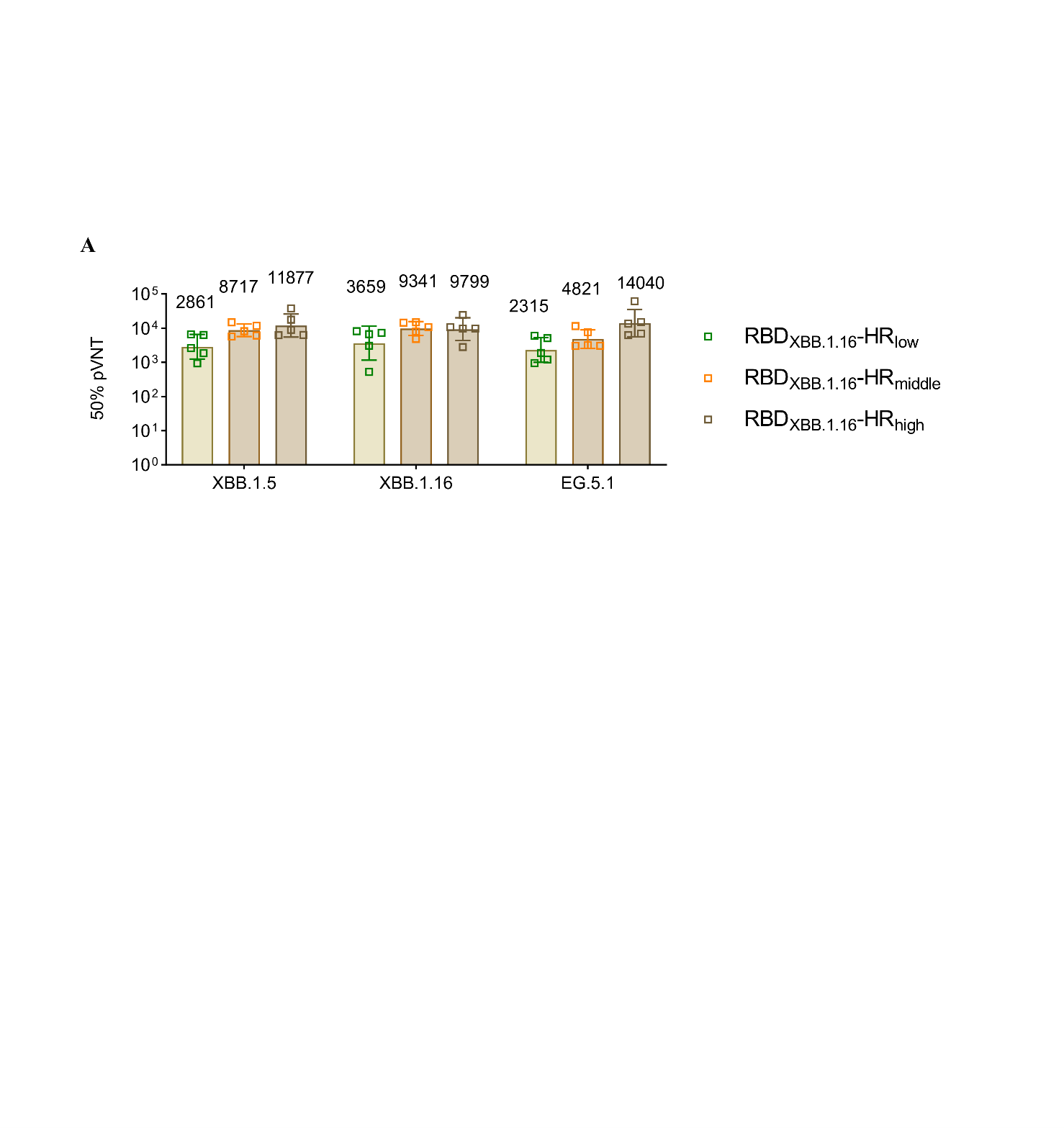


**S1.** The GMTs of naturalizing antibodies against XBB.1.5, XBB.1.16, and EG.5.1 pseudoviruses for different doses of RBD_XBB.1.16_-HR vaccine. Serum samples were collected on day 35.


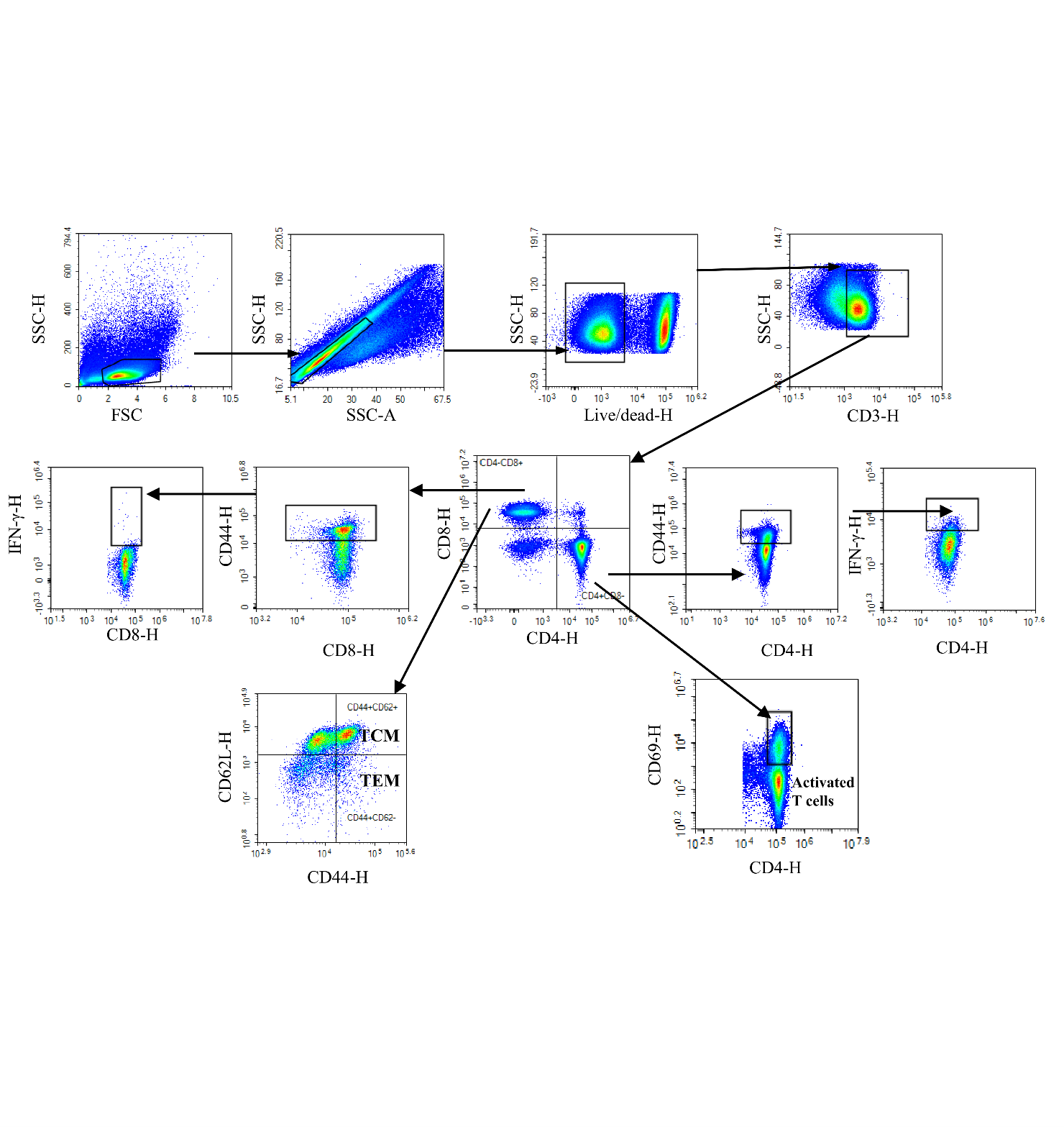


**S2.** The gating strategy for TCM, TEM, activated T cells, and cellular cytokine-secreting T cells.


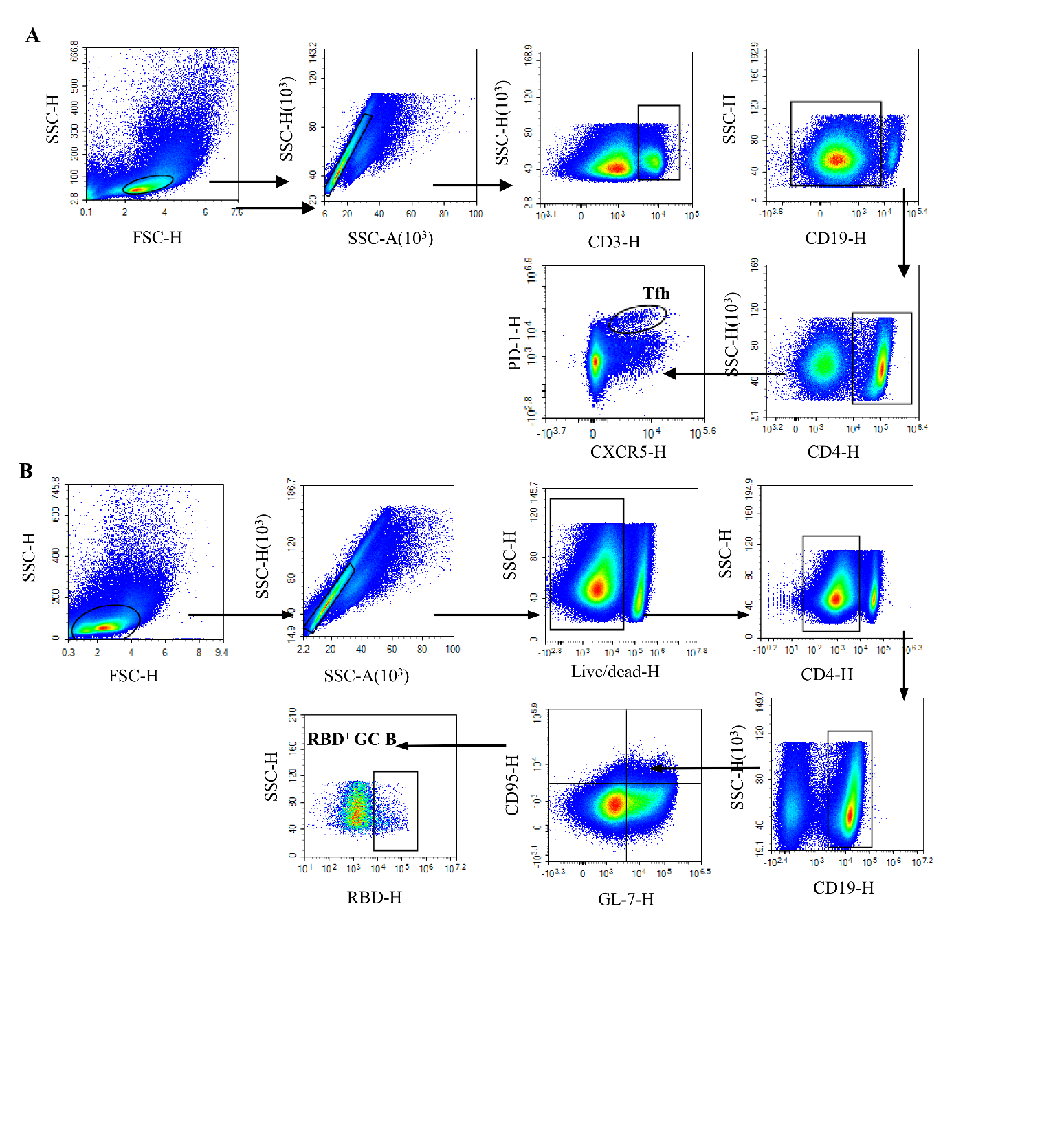


**S3**. The gating strategy for Tfh cells and RBD-specific GC B cells


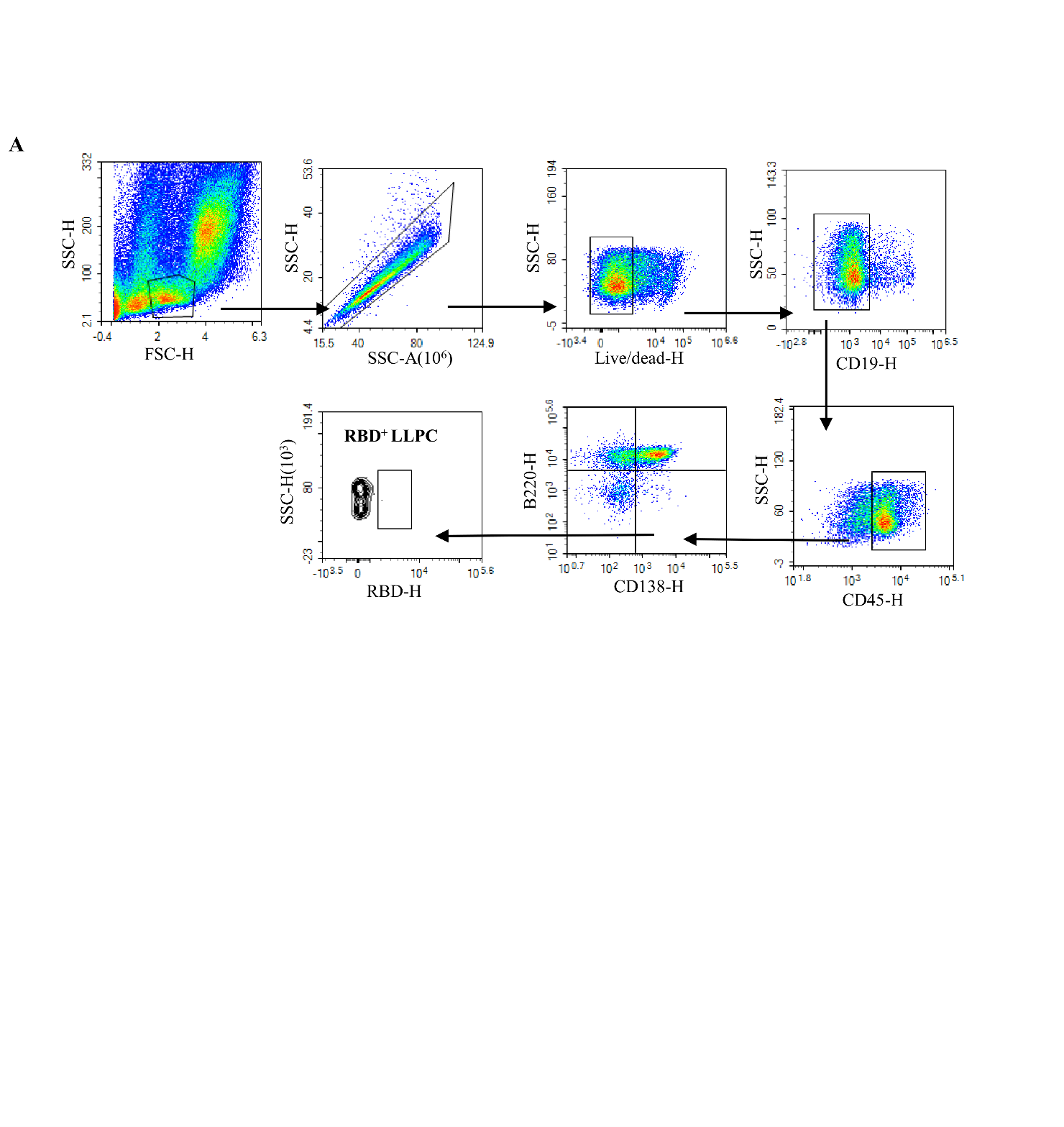


**S4**. The gating strategy for RBD-specific LLPCs.
